# Trends and predictors of optimal breastfeeding among children 0–23 months, South Asia: Analysis of national survey data

**DOI:** 10.1111/mcn.12698

**Published:** 2018-11-29

**Authors:** Rukundo K. Benedict, Hope C. Craig, Harriet Torlesse, Rebecca J. Stoltzfus

**Affiliations:** ^1^ Division of Nutritional Sciences Cornell University Ithaca New York USA; ^2^ The DHS Program ICF International Rockville Maryland USA; ^3^ UNICEF, Regional Office for South Asia Kathmandu Nepal

**Keywords:** continued breastfeeding, early initiation of breastfeeding, exclusive breastfeeding, optimal breastfeeding, prelacteal feeding, South Asia

## Abstract

Optimal breastfeeding practices, including early initiation of breastfeeding (EIBF) within 1 hr of birth, exclusive breastfeeding (EBF) for the first 6 months of age, and continued breastfeeding (CBF) for 2 years of age or beyond with appropriate complementary foods, are essential for child survival, growth, and development. Breastfeeding norms differ within and between countries in South Asia, and evidence is needed to inform actions to protect, promote, and support optimal practices. This study examines time trends and predictors of EIBF, avoidance of prelacteal feeding (APF), EBF, and CBF to 2 years using survey data from Afghanistan, Bangladesh, India, Nepal, and Pakistan since 1990. EIBF, APF, and EBF increased in Bangladesh, India, and Nepal from 1990 to 2016. EIBF and EBF increased in Pakistan from 1990 to 2013, but both EIBF and APF decreased in recent years. In Afghanistan, EIBF, APF, and EBF decreased from 2010 to 2015. CBF remained fairly constant across the region although prevalence varied by country. Significant (*p* < 0.05) predictors of suboptimal practices included caesarian delivery (4–25%), home delivery, small size at birth, and low women's empowerment. Wealth, ethnic group, and caste had varied associations with breastfeeding. Progress towards optimal breastfeeding practices is uneven across the region and is of particular concern in Afghanistan and Pakistan. There are some common predictors of breastfeeding practices across the region, however country‐specific predictors also exist. Policies, programs, and research should focus on improving breastfeeding in the context of women's low empowerment and strategies to support breastfeeding of infants born small or by caesarian section, in addition to country‐specific actions.

**Key messages**

- The generally positive trends in EIBF, APF, and EBF in Bangladesh, India, and Nepal are encouraging, but progress towards improved practices is regressing in Pakistan and Afghanistan.
- Caesarean delivery is a common predictor of suboptimal EIBF across all countries. Home delivery, small size at birth, and low women's empowerment were also associated with suboptimal breastfeeding.
- Creating a supportive breastfeeding environment can improve breastfeeding practices in South Asia. Action is required to address the child‐, maternal‐, and household‐level disparities in breastfeeding practices.

## 1 INTRODUCTION

Despite the well‐established benefits of breastfeeding on survival, health, and development, many infants are not breastfed according to World Health Organization and United Nations Children's Fund (UNICEF) recommendations on optimal breastfeeding practices (United Nations Children's Fund, 2016; World Health Organization, 2003). These practices include early initiation of breastfeeding (EIBF) within 1 hr of birth, exclusive breastfeeding (EBF) for the first 6 months of life, and continued breastfeeding (CBF) up to 2 years of age or beyond with appropriate complementary foods (World Health Organization, 2003). Avoidance of prelacteal feeding (APF) during the first 3 days of life also promotes optimal breastfeeding practices. The benefits of breastfeeding for children up to 2 years of age include lower child morbidity and mortality, higher intelligence scores and academic performance, and lower risk of ovarian and breast cancers among mothers (Victora et al., 2016). Breastfeeding could save more than 800,000 child lives and add more than $300 billion to the global economy if scaled up to near universal levels (Rollins et al., 2016; Victora et al., 2016).

South Asia is home to 26% of the world's children under 5 years of age (United Nations Children's Fund, 2016). Although the region is making progress on child nutrition, including infant feeding, there is much heterogeneity among and within countries in the region with regard to breastfeeding practices. In order to inform policies and programs to reach the global target of the World Health Assembly on EBF by 2025 and improve other breastfeeding practices, a deeper understanding of this heterogeneity is needed (World Health Organization, 2014).

Breastfeeding is influenced by multiple environments, that is, individual, family/household, community, workplace, health systems, and policy (Rollins et al., 2016; United Nations Children's Fund, 2016). Many researchers and practitioners have used a social–ecological perspective to describe how individual breastfeeding behaviours are subject to environmental factors, such as family support, community norms, and health system policies (Bronfenbrenner, 1979). This study describes the epidemiology of optimal breastfeeding between 1990 and 2016 in five countries in South Asia, which account for 99% of the region's population: Afghanistan, Bangladesh, India, Nepal, and Pakistan. The study describes trends in breastfeeding practices in South Asia since 1990 and uses a socioecological approach to identify factors associated with optimal practices in each country. We discuss the policy and program implications of the findings for these countries.

## 2 METHODS

### 2.1 Data sources

Datasets from nationally representative surveys in Afghanistan, Bangladesh, India, Nepal, and Pakistan conducted between 1990 and 2016 were used in the analysis, including Demographic Health Surveys (DHS) and National Family Health Surveys (NFHS), and Multiple Indicator Cluster Surveys (MICS). The surveys include indicators on maternal and child health and are typically conducted every 3–5 years. Depending on the country and survey round, sample sizes ranged from 5,000 to over 90,000 households.

All surveys collected data from women 15–49 years of age. Breastfeeding data were collected from women with a live birth in the 2 years preceding the interview date. We restricted our sample to include only the last‐born children living with their mother at the time of the interview. All surveys used a modified 24‐hr recall of infant feeding practices. Further information about national survey methodology and sampling procedures are available in the respective DHS, NFHS, and MICS reports.

### 2.2 Data collection

#### 2.2.1 Outcomes

The following breastfeeding outcomes were examined: EIBF, defined as the proportion of children born in the last 24 months who were put to the breast within 1 hr of birth; APF, the proportion of children aged 0–23 months who did not receive any food/drink other than breast milk during the first 3 days following delivery; EBF, the proportion of infants aged 0–5 months who received only breast milk, during the previous day; and CBF at 2 years, the proportion of children aged 20–23 months who received breast milk during the previous day (World Health Organization, 2008).

#### 2.2.2 Independent variables

**Figure 1:**
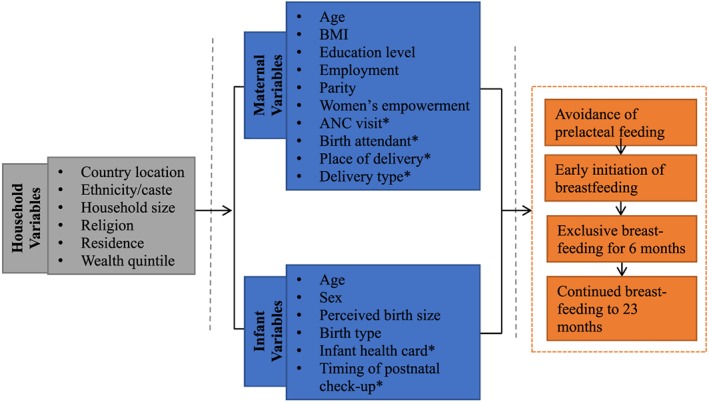
Conceptual framework on breastfeeding practices in South Asia. *We grouped health systems related variables, ANC visit, birth attendant, place of delivery, and delivery type and infant health card and postnatal check‐up, at the maternal and child level, respectively

Covariates from the child‐, maternal‐, and household‐level were included based on a UNICEF conceptual framework for breastfeeding and other reviews (Figure 1; Rollins et al., 2016; United Nations Children's Fund, 2016). Child‐level variables included sex (male/female), perceived birth size (larger‐than‐average, average, smaller‐than‐average), birth type (singlet/multiple), infant health card (yes/no), and timing of the postnatal check‐up (≤2 days, >2 days, none). Maternal‐level variables included maternal age (15–19 years, 20–29 years, ≥30 years), maternal education level (no school, primary, secondary, or more), current employment status (yes/no), maternal body mass index (BMI 1Anthropometry was not collected in Afghanistan.; underweight [<18.5 kg/m^2^], normal [18.5–24.9 kg/m^2^], overweight [≥25 kg/m^2^]), parity (low/high based on population median), place of delivery (home/health facility), birth attendant (heath professional, traditional birth attendant, and nonskilled other), and delivery type (caesarean/normal). Decision‐making autonomy (low/high) was also included, which captures women's responses to statements about categories of decisions that they make jointly or alone. Women with lower autonomy do not participate in one or more decisions, and women with high autonomy participate in all decisions. Attitudes regarding gender roles (conforming/nonconforming) capture women's responses to statements about the acceptability of wife beating in different scenarios. Conforming women agree with at least one statement, and nonconforming women do not agree with all statements. Household‐level variables included household size (categories based on average household size in country), wealth quintile (the survey wealth index, which is based on household size, water source, type of toilet, primary cooking methods, materials used for housing construction, and ownership of assets), religion (most common religion in country/other), caste in Nepal (relatively advantaged, relatively disadvantaged, disadvantaged, others), caste in India (otherwise backward caste, scheduled caste [SC], scheduled tribe [ST], others), ethnicity in Afghanistan (Pashtun, Tajik, others), and residence (urban/rural).

### 2.3 Data analyses

Breastfeeding trends analyses used data from two surveys in Afghanistan, seven surveys in Bangladesh, four surveys in India, five surveys in Nepal, and three surveys in Pakistan (Table 1). Breastfeeding outcomes (EIBF, APF, EBF, CBF) were expressed as dichotomous variables. All statistical analyses were conducted using Stata 14.0 (StataCorp. 2015. Stata Statistical Software: Release 14. College Station, TX: StataCorp LP) with the *svy* command to allow for the cluster sampling design of the surveys. Probability weights were applied to achieve nationally representative samples, and all sample sizes and proportions reported are based on these weights. The proportion of women reporting the breastfeeding practices was calculated separately for each country and for each survey round. To account for differences across surveys, nonoverlapping confidence intervals (CIs) were used to identify statistically significant differences. However, this approach is not conclusive for overlapping CIs, and significant differences may be underestimated (Knezevic, 2008). Values presented for the trends analyses are proportions with 95% CI.

**Table 1:** Survey datasets and sample size included in the trend analyses

Country	Survey	Year	Sample size^a^ (*n*)
Afghanistan	MICS	2010	4,654
	DHS	2015	11,539
Bangladesh	DHS	1993–1994	2,508
		1996–1997	2,359
		1999–2000	2,717
		2004	2,616
		2007	2,296
		2011	3,265
		2014	3,206
India	NFHS	1992–1993	21,047
		1998–1999	21,259
		2005–2006	24,821
		2015–2016	94,104
Nepal	DHS	1996	2,835
		2001	2,668
		2006	1,988
		2011	2,031
		2016	1,978
Pakistan	DHS	1991	2,631
		2007	3,376
		2012–2013	4,247

*Note*. DHS: Demographic Health Surveys; MICS: Multiple Indicator Cluster Surveys; NFHS: National Family Health Surveys.

a Sample includes mothers with a living child born in the 2 years preceding the survey.

**Figure 2:**
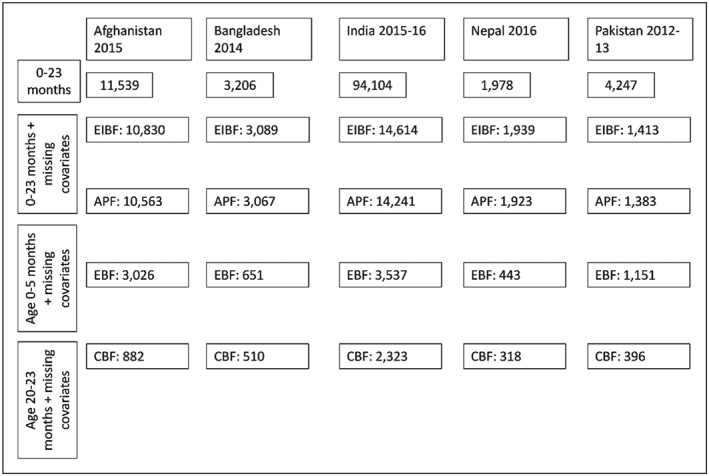
Analytic sample for final models for early initiation of breastfeeding (EIBF), avoidance of prelacteal feeding (APF), exclusive breastfeeding (EBF), and continued breastfeeding (CBF) at 2 years, South Asia. Maternal‐level variables for anthropometry (Nepal) and women's autonomy variables (India) were collected in a subsample of women. Maternal body mass index was omitted from regressions in Nepal because the total sample size was significantly reduced for all breastfeeding outcomes

Child‐, maternal‐, and household‐level variables were regressed on EIBF, APF, EBF, and CBF at 2 years in separate models for each outcome (Figure 2). Multiple logistic regression models used most recent datasets from Afghanistan (Afghanistan Demographic Health Survey (ADHS) 2015), Bangladesh (Bangladesh Demographic Health Survey (BDHS) 2014), India (National Family Health Survey (NFHS‐4) 2015–2016), Nepal (Nepal Demographic Health Survey (NDHS) 2016), and Pakistan (Pakistan Demographic Health Survey (PDHS) 2012–2013), and analyses were restricted to complete cases. Regression models were constructed using a manual stepwise backwards elimination approach to identify factors associated with the outcomes. All variables 2In Nepal, maternal BMI was excluded as it was included in a subsample of women and significantly reduced the sample size for all breastfeeding outcome analyses. were entered into the initial model, and independent variables were eliminated in stages beginning with the child‐level, maternal‐level, and household‐level last. Variables were retained in the model based on conceptual relevance and *p* value <0.15 to ensure we kept any potential meaningful confounders (Bursac, Gauss, Williams, & Hosmer, 2008). We assessed multicollinearity and omitted collinear variables. Hosmer–Lemeshow goodness‐of‐fit indices were used to assess model fit. Final models are country specific, and significant regression results are presented only for adjusted models as odds ratios with 95% CI and significance at the *p* < 0.05 level. Full model estimates are included in supplementary materials (Table S1‐S4).

### 2.4 Ethics

More information about the survey designs and data collection methods are available in the survey reports. Permission to use the data was obtained from ICF International (Rockville, Maryland) and UNICEF (New York, New York).

## 3 RESULTS

### 3.1 Trends

**Figure 3:**
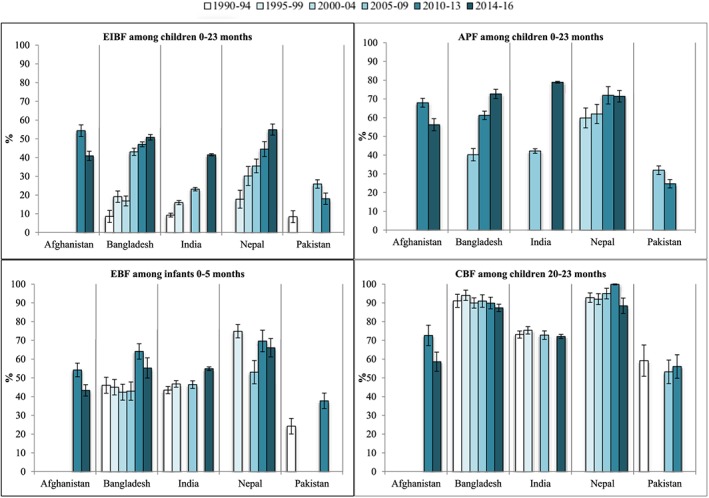
Panel of breastfeeding trends in South Asia, 1990–2016. Error bars represent the 95% confidence interval. avoidance of prelacteal feeding (APF) trends show 2000–2016 data due to limited data availability on APF in earlier surveys. From 2000 to 2004, two BDHS were released, the BDHS 1999–00 and BDHS 2004; however, only BDHS 2004 is reported in the figure above. CBF: continued breastfeeding; EBF: exclusive breastfeeding; EIBF: early initiation of breastfeeding

Surveys published in 1990–1994, 1995–1999, 2000–2004, 2005–2009, and 2010–2013 are grouped respectively, and, when available, most recent surveys (2014–2016) are presented separately (Figure 3). Samples included mothers with a living child born in the 2 years preceding the survey (Table 1).

EIBF increased between the first and last survey in all countries except Afghanistan. Between 1990–1994 and 2014–2016, EIBF increased significantly by 42 percentage points (pp) in Bangladesh, 37 pp in Nepal, and 32 pp in India. In Pakistan, EIBF increased significantly from 8.4% (95% CI [6.5, 10.8]) in 1990–1994 to 25.9% (95% CI [23.8, 28.1]) in 2005–2009 but subsequently decreased to 18.0% (95% CI [15.9, 20.3]) in 2010–2013. EIBF prevalence was highest in Afghanistan in 2010–2013 at 54.3% (95% CI [51.4, 57.5]) but decreased significantly to 40.9% (95% CI [38.4, 43.4]) by 2014–2016.

APF increased between the first and last survey in all countries except Afghanistan and Pakistan. From 2000–2004 to 2014–2016, APF increased significantly by 12 pp in Nepal from 59.8% (95% CI [54.4, 65.0]) to 71.4% (95% CI [68.3, 74.5]). In India and Bangladesh, the proportion of APF significantly increased by more than 30 pp from 2005–2009 to 2014–2016. In Pakistan, the proportion of APF significantly decreased from 31.9% (95% CI [29.7, 34.3]) to 24.7% (95% CI [22.6, 27.1]) between 2005–2009 and 2010–2013. This is consistent with the decrease in the proportion of EIBF in Pakistan during the same time period. The proportion of APF also decreased in Afghanistan from 68.0% (95% CI [65.6, 70.3]) in 2010–2013 to 56.2% (95% CI [53.0, 59.4] in 2014–2016.

EBF increased between the first and last survey in all countries except Afghanistan, Nepal, and Pakistan. In Afghanistan, EBF was 54.2% (95% CI [50.5, 57.8]) in 2010–2013 but significantly decreased by 10 pp to 43.3% (95% CI [40.3, 46.4]) by 2014–2016. EBF in Bangladesh remained close to 45% from 1995–2009, but recent trends are inconsistent. By 2010–2013, EBF in Bangladesh increased by 19 pp to 64.1% (95% CI [60.0, 68.2]) yet decreased in 2014–2016 to 55.3% (95% CI [48.8, 61.6]). In India, EBF also remained around 45% from 1995 to 2009, before increasing significantly to 55.0% (95% CI [54.1, 56.0]) by 2015–2016. In Nepal, EBF trends varied over time. From 1990–1994 to 2005–2009, EBF in Nepal decreased significantly by 22 pp from 74.9% (95% CI [71.1, 78.3]) to 53.0% (95% CI [46.8, 59.2]), then increased to 69.6% (95% CI [63.6, 75.1]) in 2010–2013 and decreased again to 66.1% (95% CI [60.8, 71.0]) in 2014–2016. In 1990–1994, less than a quarter of children 0–5 months were exclusively breastfed in Pakistan (24.2% [95% CI [20.3, 28.6]]). This proportion increased significantly by 14 pp to 37.8% (95% CI [33.7, 42.0]) in 2010–2013. Results of EBF trends disaggregated by age group are included in supplementary material (Figure S1).

CBF at 2 years was consistent between the first and last survey in all countries except Afghanistan where it decreased. From 1990–1994 to 2014–2016, CBF at 2 years remained over 85% in both Bangladesh and Nepal and over 72% in India. Over the same period in Pakistan, CBF at 2 years remained lower at proportions below 60%. In Afghanistan, recent trends in CBF at 2 years show a decrease from 71.6% (95% CI [66.8, 77.6]) in 2010–2013 to 58.6% (95% CI [53.4, 63.2]) in 2014–216.

#### 3.1.1 Multiple regressions

Table 2 summarizes select characteristics of the analytic sample across the five countries. Between 21% and 28% of children were under 6 months of age, the majority (≥99%) of children were singleton births, 12–25% of children were perceived to be smaller than average at birth, and most children did not receive a postnatal check‐up within 2 days of birth. Among maternal‐level variables, 5–25% of women were adolescents, facility births ranged from 40% to 85%, caesarean section deliveries ranged from 4% to 25%, and 21–55% of women had four or more ANC visits. Household wealth quintiles were distributed between richest (14–20%) and poorest (17–23%).

**Table 2:** Select child (0–23 months), maternal, and household analytic sample characteristics

Characteristics	Afghanistan, DHS 2015	Bangladesh, DHS 2014	India, DHS 2015–16	Nepal, DHS 2016	Pakistan, DHS 2012–13
	*N* = 10, 830	*N* = 3089	*N* = 14,614	*N* = 1,939	*N* = 1,413
	%	%	%	%	%
Child characteristics					
Child age (months)					
0–6	27.9	21.1	23.1	22.9	28.3
6–8	14.1	12.9	14.3	12.1	11.6
9–11	10.2	14.6	12.9	13.6	14.5
12–17	32.4	27.0	25.8	25.9	30.1
18–23	15.4	24.4	23.9	25.5	15.5
Birth size					
Larger than average	14.3	11.6	20.37	14.9	6.6
Average	60.5	68.4	67.7	68.1	72.1
Smaller than average	25.1	20.1	11.9	17.1	21.3
Birth type					
Singlet	99.1	99.6	99.3	99.3	99.2
Multiple	0.9	0.4	0.7	0.7	0.8
Postnatal check‐up					
None	75.1	35.6	61.1	63.9	48.9
≤2 days	9.5	55.0	28.9	10.2	41.9
>2 days	15.4	9.4	10.0	25.9	9.2
Maternal characteristics					
Maternal age (years)					
15–19	6.3	24.5	5.7	14.6	4.9
20–29	61.2	58.8	75.8	67.7	59.2
30+	32.5	16.8	18.5	17.7	35.9
Antenatal care (ANC) visit					
0–3	78.8	68.5	44.2	28.5	60.1
4+	21.2	31.5	54.8	71.5	39.9
Delivery type					
Cesarean	3.7	24.6	20.6	10.1	16.1
Normal	96.3	75.4	79.4	89.9	83.9
Place of delivery					
Home	43.8	60.1	15.4	34.3	46.5
Health facility	56.2	39.9	84.6	65.7	53.5
Household characteristics					
Wealth quintile					
Richest	20.0	19.4	16.3	14.6	18.0
Richer	21.4	19.5	18.4	20.5	22.2
Middle	21.3	20.3	21.2	22.9	19.4
Poorer	19.6	19.0	20.9	21.0	20.1
Poorest	17.7	21.8	23.2	20.9	20.3

*Note*. DHS: Demographic and Health surveys.

##### Early initiation of breastfeeding

Barriers to EIBF included cesarean delivery in all countries; smaller perceived birth size in Afghanistan, India, and Nepal; low decision‐making autonomy in India and Pakistan; gender‐conforming attitudes and maternal overweight in Pakistan; medium household size in India; birth assistance from a traditional birth attendant and other castes in Nepal (Table 3a). Facilitators to EIBF included higher maternal education and attending four or more ANC visits in India and the Tajik and other ethnicities in Afghanistan.

**Table 3a:** Adjusted logistic regression results for predictors of EIBF and APF using latest available data

Variables	EIBF among infants 0–23 monthsOR (95% CI)					APF among infants 0–23 monthsOR (95% CI)				
	Afghanistan 2015 (*n* = 10,830)	Bangladesh 2014 (*n* = 3,089)	India 2015–2016 (*n* = 14,614)	Nepal 2016 (*n* = 1,939)	Pakistan 2012–2013 (*n* = 1,413)	Afghanistan 2015 (*n* = 10,563)	Bangladesh 2014 (*n* = 3,067)	India 2015–2016 (*n* = 14,241)	Nepal 2016 (*n* = 1,923)	Pakistan 2012–2013 (*n* = 1,383)
Child variables										
Birth size										
Larger than average	1.00		1.00			1.00		1.00		1.00
Average	0.87 (0.73–1.04)		0.96 (0.84–1.09)	0.82 (0.61–1.11)		0.96 (0.76–1.22)		0.76** (0.66‐0.89)		0.57* (0.33‐0.98)
Smaller than average	0.64** (0.51–0.80)		0.82* (0.69‐0.98)	0.67* (0.45‐1.00)		0.68* (0.51‐0.89)		0.54** (0.44‐0.66)		0.57 (0.30–1.10)
Postnatal check‐up										
None	1.00	1.00	1.00		1.00	1.00	1.00			
≤2 days	0.71** (0.59–0.86)	1.29* (1.03‐1.62)	1.17* (1.04‐1.32)		0.64 (0.41–1.00)	0.71* (0.55‐0.90)	0.69* (0.55‐0.85)			
>2 days	0.96 (0.80–1.14)	1.16 (0.81–1.68)	1.26* (1.08‐1.49)		0.33* (0.17‐0.62)	0.61** (0.49‐0.75)	1.13 (0.73–1.74)			
Maternal variables										
ANC visit										
0–3			1.00							
4+			1.63** (1.46‐1.82)							
Birth attendant										
Nonskilled others				1.00			1.00		1.00	
Traditional birth attendant				0.52* (0.27‐0.98)			1.10 (0.77–1.58)		0.37* (0.20‐0.69)	
Health professional				1.13 (0.72–1.76)			1.60* (1.18‐2.18)		1.20 (0.85–1.70)	
Current employment										
No									1.00	
Yes									1.54* (1.15‐2.05)	
Decision‐making autonomy										
High			1.00		1.00	1.00		1.00		
Low			0.88* (0.80‐0.97)		0.67* (0.46‐0.96)	0.82* (0.68‐0.99)		0.83* (0.74‐0.94)		
Delivery type										
Not caesarean	1.00	1.00	1.00	1.00	1.00		1.00	1.00	1.00	
Cesarean	0.42** (0.27–0.68)	0.32** (0.23‐0.44)	0.53** (0.46‐0.60)	0.15** (0.10‐0.23)	0.40* (0.19‐0.81)		0.40** (0.29‐0.56)	0.49** (0.42‐0.57)	0.26** (0.17‐0.41)	
Gender role attitudes										
Nonconforming					1.00	1.00				
Conforming					0.66* (0.46‐0.96)	0.77* (0.64‐0.94)				
Maternal BMI										
Normal					1.00					
Underweight					0.70 (0.39–1.26)					
Overweight					0.59* (0.40‐0.89)					
Maternal education										
No school			1.00					1.00	1.00	
Primary			1.05 (0.89–1.24)					1.19 (0.99–1.43)	1.38 (0.87–2.18)	
Secondary+			1.19* (1.05‐1.35)					1.43** (1.23‐1.66)	1.71* (1.22‐2.41)	
Place of delivery										
Home		1.00	1.00	1.00						
Health facility		0.76* (0.58–0.98)	1.41** (1.22‐1.63)	1.80* (1.18‐2.76)						
Household variables										
Caste										
India										
Otherwise backwards caste								1.00		
Scheduled caste								1.21* (1.05‐1.39)		
Scheduled tribe								2.25** (1.82‐2.79)		
Other								1.09 (0.93–1.27)		
Nepal										
Relatively Advantaged				1.00					1.00	
Relatively Disadvantaged				0.96 (0.71–1.30)					0.71 (0.50–1.00)	
Disadvantaged				0.87 (0.60–1.26)					0.74 (0.52–1.17)	
Other				0.69* (0.49‐0.98)					0.64* (0.45‐0.91)	
Ethnicity										
Afghanistan										
Pashtun	1.00									
Tajik	1.65** (1.26‐2.09)									
Other	1.56** (1.21‐1.91)									
Household size (members)										
India										
0–5			1.00					1.00		
6–9			0.89* (0.80‐0.99)					0.84* (0.73‐0.96)		
10+			0.88 (0.77–1.01)					0.69** (0.59‐0.81)		
Wealth quintile										
Richest	1.00		1.00		1.00			1.00	1.00	1.00
Richer	1.37* (1.12‐1.80)		1.24* (1.04‐1.47)		0.36* (0.19‐0.68)			1.25* (1.02‐1.55)	1.18 (0.79–1.77)	0.62* (0.38‐1.00)
Middle	1.32* (1.11‐1.77)		1.41** (1.16‐1.70)		0.66 (0.35–1.26)			1.19 (0.98–1.45)	1.26 (0.80–1.98)	0.64 (0.39–1.06)
Poorer	1.26* (1.08‐1.72)		1.42** (1.17‐1.73)		0.58 (0.31–1.10)			1.30* (1.06‐1.58)	1.95* (1.26‐3.01)	0.82 (0.49–1.39)
Poorest	1.31* (1.16‐1.88)		1.46* (1.17‐1.81)		1.11(0.54–2.31)			1.07 (0.86–1.33)	3.60** (2.04‐6.35)	1.57 (0.89–2.75)

*Note*. APF: avoidance of prelacteal feeding; BMI: body mass index; EIBF: early initiation of breastfeeding. Sample sizes are different because models differed by country and by outcome. Final models for EIBF included birth size, postnatal check‐up, place of delivery, delivery type, gender role attitudes, household size, wealth quintile, and ethnicity (Hosmer–Lemeshow goodness‐of‐fit 0.520; Afghanistan); postnatal check‐up, place of delivery, and gender role attitudes (Hosmer–Lemeshow goodness‐of‐fit 0.994; Bangladesh); birth size, postnatal check‐up, maternal education, ANC visit, place of delivery, delivery type, decision‐making autonomy, residence, household size, and wealth quintile, caste (Hosmer–Lemeshow goodness‐of‐fit 0.703; India); birth size, place of delivery, delivery type, residence, wealth quintile, and caste (Hosmer–Lemeshow goodness‐of‐fit 0.967; Nepal); and postnatal check‐up, maternal education, maternal BMI, delivery type, decision‐making autonomy, and wealth quintile (Hosmer–Lemeshow goodness‐of‐fit 0.954; Pakistan). Final models for APF included birth size, postnatal check‐up, current employment, gender role attitudes, decision‐making autonomy, and ethnicity (Hosmer–Lemeshow goodness‐of‐fit 0.879; Afghanistan); postnatal check‐up, place of delivery, delivery type, and residence (Hosmer–Lemeshow goodness‐of‐fit 0.997; Bangladesh); birth size, maternal education, decision‐making autonomy, delivery type, household size, and caste (Hosmer–Lemeshow goodness‐of‐fit 0.992; India); maternal age, maternal education, delivery type, household size, wealth quintile, and caste (Hosmer–Lemeshow goodness‐of‐fit 0.797; Nepal); and birth size, maternal BMI, delivery type, household size, and wealth quintile (Hosmer–Lemeshow goodness‐of‐fit 0.548; Pakistan).

* *p* < 0.05.

** *p* < 0.001**.**

**Table 3b:** Adjusted logistic regression results for predictors of EBF and CBF using latest available data

Variables	EBF among infants 0–5 months OR (95% CI)					CBF among infants 20–23 months OR (95% CI)				
	Afghanistan 2015 (*n* = 3,026)	Bangladesh 2014 (*n* = 651)	India 2015–2016 (*n* = 3,537)	Nepal 2016 (*n* = 443)	Pakistan 2013 (*n* = 1,151)	Afghanistan^a^ 2015 (*n* = 882)	Bangladesh^a^ 2014 (*n* = 510)	India 2015–2016 (*n* = 2,323)	Nepal^a^ 2016 (*n* = 318)	Pakistan 2013 (*n* = 396)
Child variables										
Birth size										
Larger than average	1.00									
Average	1.03 (0.67–1.57)									
Smaller than average	0.55* (0.35–0.86)									
Child age (months)										
0–1	2.84** (2.08‐3.88)	6.46** (3.22–12.97)	3.98** (3.03–5.22)	6.13** (3.43–10.93)	3.90** (2.56–5.94)					
2–3	1.55* (1.17‐2.06)	3.27** (1.77–6.04)	2.30** (1.85–2.87)	3.93** (2.15–7.17)	2.06** (1.39–3.05)					
4–5	1.00	1.00	1.00	1.00	1.00					
Child sex										
Male			1.00							
Female			0.80* (0.66‐0.97)							
Infant health card										
No		1.00	1.00							
Yes		0.55* (0.33‐0.91)	1.54* (1.21‐1.97)							
Maternal variables										
Birth attendant										
Nonskilled others	1.00									
Traditional birth attendant	0.43** (0.29–0.63)									
Health professional	0.60* (0.43–0.83)									
Decision‐making autonomy										
High										1.00
Low										0.51* (0.27‐0.93)
Delivery type										
Not caesarean					1.00					
Cesarean					0.55* (0.36‐0.85)					
Gender role attitudes										
Nonconforming	1.00									
Conforming	0.68* (0.47‐0.99)									
Maternal age (years)										
15–19			1.00							
20–29			0.62* (0.42‐0.94)							
30+			0.66 (0.41–1.03)							
Maternal BMI										
Normal								1.00		
Underweight								1.57* (1.13‐2.18)		
Overweight								0.86 (0.60–1.24)		
Maternal education										
No school										1.00
Primary										2.36* (1.03‐5.42)
Secondary+										1.51 (0.76–3.01)
Household variables										
Caste										
India										
Otherwise backwards caste			1.00							
Scheduled caste			1.14 (0.90–1.46)							
Scheduled tribe			1.39* (1.04–1.86)							
Other			0.75 (0.53–1.05)							
Ethnicity										
Afghanistan										
Pashtun	1.00									
Tajik	0.56** (0.41‐0.77)									
Other	0.39** (0.28‐0.56)									
Household size (members)										
Bangladesh										
0–4		1.00								
5–8		0.47* (0.27‐0.84)								
9+		0.55 (0.30–1.03)								
Wealth quintile										
Richest				1.00				1.00		1.00
Richer				1.17 (0.53–2.59)				1.23 (0.79–1.92)		1.23 (0.54–2.76)
Middle				2.17* 1.04–4.53)				1.70 (1.10–2.60)		3.94* (1.49‐10.44)
Poorer				1.19 (0.52–2.68)				2.42** (1.57‐3.74)		3.18* (1.19‐8.46)
Poorest				2.09 (0.99–4.42)				3.27** (2.09‐5.12)		5.27* (1.76‐15.73)

*Note*. BMI: body mass index; CBF: continued breastfeeding; EBF: exclusive breastfeeding. Sample sizes are different because models differed by country and by outcome. Final models for EBF included child age, birth size, postnatal check‐up, ANC visit, gender role attitudes, wealth quintile, and ethnicity (Hosmer–Lemeshow goodness‐of‐fit 0.985; Afghanistan); child age, infant health card, and household size (Hosmer–Lemeshow goodness‐of‐fit 0.988; Bangladesh); child age, child sex, infant health card, maternal age, and caste (Hosmer–Lemeshow goodness‐of‐fit 0.430; India); child age, decision‐making autonomy, wealth quintile, and caste (Hosmer–Lemeshow goodness‐of‐fit 0.656; Nepal); child age, maternal age, and delivery type (Hosmer–Lemeshow goodness‐of‐fit 0.992; Pakistan). Final models for CBF included parity, household size, and ethnicity (Hosmer–Lemeshow goodness‐of‐fit 0.688; Afghanistan); maternal age, maternal BMI, and wealth quintile (Hosmer–Lemeshow goodness‐of‐fit 0.892; Bangladesh); maternal BMI, decision‐making autonomy, wealth quintile, and caste (Hosmer–Lemeshow goodness‐of‐fit 0.925; India); current employment, gender role attitudes, and residence (Hosmer–Lemeshow goodness of fit 0.999; Nepal); child sex, maternal education, parity, decision‐making autonomy, and wealth quintile (Hosmer–Lemeshow goodness‐of‐fit 0.777; Pakistan).

a No significant predictors of CBF 20–23 months were identified in Afghanistan, Bangladesh, and Nepal.

* *p* < 0.05.

** *p* < 0.001.

The direction of association was not consistent by country for several variables. Significant associations included child postnatal check‐up within 2 days of delivery in Afghanistan, Bangladesh, and India; postnatal check‐up more than 2 days after delivery in India and Pakistan; health facility delivery in Bangladesh, India, and Nepal; and wealth quintile in Afghanistan, India, and Pakistan.

##### Avoidance of prelacteal feeding

Barriers to APF included average or smaller than average birth size in Afghanistan, India, and Pakistan; cesarean delivery in Bangladesh, India, and Nepal; child postnatal check‐up within 2 days of delivery or after 2 days of delivery in Afghanistan and Bangladesh; low decision‐making autonomy in Afghanistan and India; other caste groups in Nepal; gender‐conforming attitudes in Afghanistan; and larger household size in India (Table 3a). Facilitators to APF included women with secondary or higher education in India and Nepal, SCs and STs in India, and current employment in Nepal. The direction of association was not consistent for birth assistance from a traditional birth attendant or health professional in Nepal and Bangladesh and wealth quintile across India, Nepal, and Pakistan.

##### Exclusive breastfeeding

Barriers to EBF included female child in India, perceived small birth size in Afghanistan, birth assistance from health professionals traditional birth attendants and gender‐conforming attitudes in Afghanistan, maternal age in India, caesarian delivery in Pakistan, medium household size in Bangladesh, and the Tajik and other ethnic groups in Afghanistan (Table b). Child age in all countries, STs in India, and middle wealth quintile in Nepal were positively associated with EBF. The direction of association was significant but not consistent for infant health card in Bangladesh and India.

##### Continued breastfeeding at 2 years

Few variables were associated with CBF across countries (Table 3b). Lower decision‐making autonomy in Pakistan was a significant barrier to CBF at 2 years. Facilitators to CBF at 2 years included maternal underweight in India, maternal primary education in Pakistan, and poorer wealth quintiles in India and Pakistan. There were no significant associations with CBF at 2 years in Afghanistan, Bangladesh, or Nepal.

## 4 DISCUSSION

This study examined trends and predictors of EIBF, APF, EBF, and CBF in five countries in South Asia. The evidence shows positive trends for EIBF, APF, and EBF in Bangladesh, India, and Nepal, but Afghanistan and Pakistan lag behind in almost all breastfeeding practices. Predictors of breastfeeding practices varied across countries; however, cesarean delivery and child age were common predictors for EIBF and EBF, respectively. Taken together, the findings summarize progress and identify predictors of EIBF, APF, EBF, and CBF for each country in the region and have implications for policy and programs.

### 4.1 Early initiation of breastfeeding

Despite steady improvements in EIBF in Bangladesh, India, and Nepal, recent declines in Afghanistan and Pakistan are a concern. Lack of health system support for EIBF and security problems in much of Afghanistan and parts of Pakistan may hinder implementation of interventions designed to support EIBF. Total EIBF prevalence for Afghanistan, Bangladesh, India, and Nepal are comparable with the global average (46%; United Nations Children's Fund, 2016). However, accelerating progress to support child health and achieve global targets is required in all countries.

Among child‐level factors, women's perception of small birth size was identified as a risk factor for delayed initiation in Afghanistan, India, and Nepal. Perceptions of small birth size could reflect low birth weight, which has been associated with poor EIBF (Pollitt, Gilmore, & Valcarcel, 1978; Sundaram et al., 2013). Actions to prevent low birth weight, which is prevalent in the region (United Nations Children's Fund, 2014), and to support the establishment of breastfeeding in low birth weight infants could increase EIBF. Postnatal check‐up, a proxy for the quality of health services in our analysis, was positively associated with EIBF in Bangladesh and India but negatively associated in Afghanistan and Pakistan. The inconsistent findings suggest that the provision of breastfeeding promotion and support by health providers may vary in coverage or quality across countries.

Caesarean delivery was associated with delayed initiation of breastfeeding in all countries. Additionally, health facility delivery was associated with delayed breastfeeding in Bangladesh but not in Nepal and India, and birth assistance from traditional birth attendants was associated with delayed breastfeeding in Nepal. Whereas in India, four or more ANC visits predicted EIBF. The results highlight the role of health workers and health facilities in supporting EIBF, and as cesarean deliveries rise in South Asia (Betran et al., 2016), it underscores the importance of ensuring policies such as Baby Friendly Hospital Initiative support EIBF in all infants, including those born by caesarian section. In Pakistan, overweight and obesity were risk factors for delayed initiation. As overweight and obesity rise in the South Asia region, more countries will need to address breastfeeding support for this population (NCD Risk Factor Collaboration, 2017). Low decision‐making autonomy was another factor associated with suboptimal EIBF in India and Pakistan, whereas higher education was a predictor of EIBF. The results are similar to a previous findings showing women's decision‐making autonomy is a predictor of EIBF in South Asia but not other breastfeeding practices (Smith, Ramakrishnan, Ndiaye, Haddad, & Matrorell, 2003). Improving the status of women in South Asia may be beneficial for EIBF, but further research is warranted.

At the household level, there were inconsistent findings for wealth in Pakistan, Afghanistan, and India, and caste and ethnicity were associated with EIBF in Nepal and Afghanistan. The results show an influence of these household‐level variables on EIBF, but the relationships are likely context dependent. These results suggest research is required to better understand and address the drivers of the behaviours specific to different castes and ethnicities.

### 4.2 Avoidance of prelacteal feeding

Prelacteal feeding is known to disrupt EIBF and EBF by delaying the onset of breastfeeding and milk arrival (Ahmed, Rahman, & Alam, 1996; Perez‐Escamilla, Segura‐Millan, Canahuati, & Allen, 1996). APF steadily rose in Bangladesh, India, and Nepal but declined recently in Afghanistan and Pakistan where rates are currently lowest. The declines may be related to sociocultural barriers and marketing of breast milk substitutes. However, in all countries, prelacteal feeding is still a problem, and common prelacteals include honey, water, and other animal milks (Figure S2). Measures to improve EIBF and EBF must continue to address prelacteal feeding.

There were similarities in predictors for EIBF and APF. At the child level, small or average perceived birth size in Afghanistan, India, and Pakistan and infant postnatal check‐ups in Afghanistan were predictors for both suboptimal EIBF and APF. Results for postnatal check‐ups in Afghanistan suggest poor quality of health services to support breastfeeding. Perceptions of breast milk insufficiency are common across cultures, and caregivers and women who perceive their infants as small, may provide prelacteals to compensate for their perceived milk insufficiency (Gatti, 2008; Sundaram et al., 2013). Small newborns may also have anatomical problems with latching and poor suckling patterns for which lactation support is required, but often these services are not available in many parts of South Asia (Gryboski, 1969).

At the maternal level, education in India and Nepal predicted both EIBF and APF, and delivery assistance from health professionals in Bangladesh predicted APF. Cesarean section delivery in Bangladesh, birth assistance from traditional birth attendants in Nepal, and low autonomy in Afghanistan and India were associated with suboptimal EIBF and APF, and in Afghanistan, nonconforming gender role attitudes predicted suboptimal APF. The findings for APF reinforce the need for breastfeeding promotion and support for mothers who deliver via caesarean section, ensuring health workers promote positive breastfeeding practices and the avoidance of prelacteals and for improving women's status. Interestingly, employment was associated with APF in Nepal and may suggest improved status of women is beneficial for APF. At the household level, inconsistencies in the relationship between APF and wealth across countries, and the significant relationship with caste in Nepal and India, further highlight the different sociocultural norms for prelacteal feeding across contexts. Prelacteal feeding is influenced by misconceptions about colostrum and local feeding customs (Ali, Ali, Imam, Ayub, & Billoo, 2011; Patel, Banerjee, & Kaletwad, 2013; Sharma & Byrne, 2016). Therefore, addressing context‐specific beliefs and behaviours are crucial.

### 4.3 Exclusive breastfeeding

EBF rates in Bangladesh, India, and Nepal were slightly above the World Health Assembly 2025 breastfeeding target of 50%; however, the recent decline in Afghanistan and low rate in Pakistan are a concern. Possible reasons for this include geographic and security barriers limiting interventions to support EBF, weak health systems, and marketing of breast milk substitutes.

Younger child age was the only child‐level predictor of EBF in all countries, whereas small birth size and female child were negatively associated with EBF in Afghanistan and India, respectively. Another study in India also reported associations between female children and shorter duration of EB (Jayachandran & Kuziemko, 2011). Cesarean delivery in Pakistan, delivery assistance from traditional birth attendants or health professionals in Afghanistan, middle‐aged mothers in India, and larger household size in Bangladesh were risk factors for suboptimal EBF. EBF was associated with ethnic groups in Afghanistan and India, illustrating the influence of local norms on EBF in these countries. The perception of insufficient milk supply and sociocultural norms (Gatti, 2008; Pries et al., 2016) are all reported barriers to EBF (Patel et al., 2015). EBF for 6 months is a challenge for women everywhere, and our results illustrate understanding local context is important for overcoming barriers to EBF. Interventions that are multicomponent, targeting women, families, communities, and health facilities can help women start and sustain EBF for 6 months (Menon et al., 2016).

### 4.4 Continued breastfeeding

Although CBF at 2 years is higher in all five countries than the global average (46%; United Nations Children's Fund, 2016), recent declines in CBF prevalence are a concern for Afghanistan and Nepal. In regions with low diet diversity, CBF helps provide children with essential nutrients, continues to offer protection from infection, and is associated with child development (United Nations Children's Fund, 2016).

Maternal‐ and household‐level predictors of CBF at 2 years included women with low BMI in India, no education in Pakistan, and poorer households in both countries. The results suggest socio‐economic disparities play a role in the maintenance of breastfeeding for 2 years with those more disadvantaged breastfeeding longer (Victora et al., 2016). Interventions to support CBF must be tailored to target women across the socioeconomic spectrum, and this will be increasingly important as countries in the region experience economic growth.

### 4.5 Implications for policy and programs

The variation in predictors of optimal breastfeeding between countries illustrate that policy and program interventions need to be based on an understanding of local determinants for suboptimal breastfeeding. In South Asia, national action for breastfeeding, including national policies and laws protecting, promoting, and supporting optimal breastfeeding, exists (Thow et al., 2017). However, our results indicate more action is required at the national and subnational levels to ensure that these polices are implemented and monitored.

At the health facility level, suboptimal breastfeeding practices among cesarean delivery births is a clear example of where action is required in all countries. All mothers, regardless of the mode of delivery, should be supported to initiate breastfeeding immediately after delivery (Prior et al., 2012). Strategies to improve breastfeeding outcomes for infants delivered by caesarian section include adoption of supportive hospital policies, training of medical staff to support breastfeeding postdelivery, education about caesarian delivery and breastfeeding, and reduction of caesarian deliveries that are not medically required (Kuyper, Vitta, & Dewey, 2014).

Low birth weight infants require special attention. Ensuring facility and community health workers are trained to support mothers to put low birth weight infants who are able to breastfeed to the breast as soon as possible after birth when they are clinically stable or to assist mothers to express breast milk or access human milk for infants that cannot be fed at the breast is essential (World Health Organization, 2011). In South Asia, India has an established network of human milk banks for low birth weight infants (Haiden & Ziegler, 2016). Further, preventing low birth weight by improving maternal nutritional status before and during pregnancy may also improve breastfeeding practices.

The health system also has a crucial role in promoting and supporting breastfeeding practices during antenatal care and throughout the first 2 years of a child's life. Our findings indicate that interventions should be based on a local understanding of social–cultural barriers, including potentially harmful traditional practices and the low autonomy of women. Interventions delivered by health workers and community‐based workers to inform, educate, and counsel on breastfeeding in the home/family, community, and health facility environments all demonstrate effectiveness, highlighting the impact of multiple supportive environments (Benedict, Craig, Torlesse, & Stoltzfus, 2018). A breastfeeding supportive environment should include not only the immediate family and health system but also the broader community.

### 4.6 Limitations

The cross‐sectional nature of the survey data limits inferences about directionality of associations in this study; however, our analyses do not make any causal claims. There is also the possibility of response bias from mothers due to the recall method used to assess breastfeeding practices, and this could affect accuracy of the estimates. In addition, social desirability bias for all self‐reported breastfeeding practices could affect accuracy of the estimates. Sample sizes for some breastfeeding practices such as CBF were small in some countries, limiting inferences. Finally, the comparability across countries may be limited by the different survey years.

## 5 CONCLUSION

Over the last 25 years, there has been a steady increase in EIBF, APF, and EBF in Bangladesh, India, and Nepal. CBF, however, has not shown the same improvement, and, in recent years, all breastfeeding practices in Afghanistan and EIBF and APF in Pakistan have declined. Our study identified child‐, maternal‐, and household‐level factors associated with suboptimal breastfeeding practices in South Asia. The most common predictors of suboptimal breastfeeding included caesarian delivery, small size at birth, home delivery, and low women's empowerment. This information can assist policy and program managers to strengthen the design and implementation of actions to protect, promote, and support breastfeeding.

## CONFLICTS OF INTEREST

The authors declare they have no conflicts of interest that would be interpreted as having influenced this research.

## CONTRIBUTIONS

RKB and HCC conducted analyses. RKB, RJS, and HT conceptualized the manuscript. The manuscript was written by RKB and edited by RKB, HCC, RJS, and HT

## Supporting information

Figure S1. Proportion of infants 0–5 months old fed exclusively with breast milk by age group in South Asia, 1990–2016.
Figure S2. Prelacteal feeds given in the first 3 days of life, among children 0–23 months living with mother, 2000–2016.
Table S1. Predictors of early initiation of breastfeeding (EIBF) within 1 hr of birth among children 0–23 months who live with their mother.
Table S2. Predictors of avoidance of prelacteal feeding (APF) among children 0–23 months who live with their mother.
Table S3. Predictors of exclusive breastfeeding (EBF) among children 0–5 months who live with their mother.
Table S4. Predictors of continued breastfeeding (CBF) among children 20–23 months who live with their mother.
