# Supplementary material for: Trends and predictors of optimal breastfeeding among children 0–23 months, South Asia: Analysis of national survey data
Source: Matern Child Nutr. 2018 Nov 29;14(Suppl 4):e12698. doi: 10.1111/mcn.12698 (PMC6519202; doi:10.1111/mcn.12698)
Supplement: Supplementary file 1 — Figure S1. Proportion of infants 0–5 months old fed exclusively with breast milk by age group in South Asia, 1990–2016. Figure S2. Prelacteal feeds given in the first 3 days of life, among children 0–23 months living with mother, 2000–2016. Table S1. Predictors of early initiation of breastfeeding (EIBF) within 1 hr of birth among children 0–23 months who live with their mother. Table S2. Predictors of avoidance of prelacteal feeding (APF) among children 0–23 months who live with their mother. Table S3. Predictors of exclusive breastfeeding (EBF) among children 0–5 months who live with their mother. Table S4. Predictors of continued breastfeeding (CBF) among children 20–23 months who live with their mother. [file MCN-14-e12698-s001.docx]

Figure S.1. Proportion of infants 0-5 months old fed exclusively with breastmilk by age group in South Asia, 1990-2016.

Error bars represent the 95% confidence interval (CI). CIs are based on exclusive breastfeeding among infants 0-5 months of age; CIs at each age group could not be calculated. Age strata within each bar show subgroups of respondents by age of infant. BDHS 1999-00 is not included. The proportion of infants who were exclusively breastfed decreased with child age with exceptions in Afghanistan 2010-13 and Nepal 1995-99. Between 1990-04 and 2014-16, little to no change was observed in EBF among children aged 4-5 months in Bangladesh, India and Pakistan. EBF among children aged 4-5 months declined slightly in Afghanistan and was inconsistent in Nepal.

Figure S.2. Prelacteal feeds given in the first three days of life, among children 0-23mo living with mother, 2000-2016.

Table S.1. Predictors of early initiation of breastfeeding (EIBF) within one hour of birth among children 0-23 months who live with their mother.

| Predictors of EIBF | Afghanistan  2015  (n=10,830) | | | Bangladesh  2014  (n=3,089) | | | India  2015-16  (n=14,614) | | | Nepal  2016  (n=1,939) | | | Pakistan  2013  (n=1,413) | | |
| --- | --- | --- | --- | --- | --- | --- | --- | --- | --- | --- | --- | --- | --- | --- | --- |
|  | OR | 95% CI | | OR | 95% CI | | OR | 95% CI | | OR | 95% CI | | OR | 95% CI | |
| ***Child variables*** |  |  |  |  |  |  |  |  |  |  |  |  |  |  |  |
| **Birth size** |  |  |  |  |  |  |  |  |  |  |  |  |  |  |  |
| Larger than average | 1.00 |  |  |  |  |  | 1.00 |  |  | 1.00 |  |  |  |  |  |
| Average | 0.87 | 0.73 | 1.04 |  |  |  | 0.96 | 0.84 | 1.09 | 0.82 | 0.61 | 1.11 |  |  |  |
| Smaller than average | 0.64^**^ | 0.51 | 0.80 |  |  |  | 0.82^*^ | 0.68 | 0.98 | 0.67^*^ | 0.45 | 1.00 |  |  |  |
| **Postnatal check-up** |  |  |  |  |  |  |  |  |  |  |  |  |  |  |  |
| None | 1.00 |  |  | 1.00 |  |  | 1.00 |  |  |  |  |  | 1.00 |  |  |
| ≤2 days | 0.71^**^ | 0.59 | 0.86 | 1.29^*^ | 1.03 | 1.62 | 1.17^*^ | 1.04 | 1.32 |  |  |  | 0.64 | 0.41 | 1.00 |
| >2 days | 0.96 | 0.80 | 1.14 | 1.17 | 0.81 | 1.68 | 1.26^*^ | 1.08 | 1.49 |  |  |  | 0.33^*^ | 0.17 | 0.62 |
| ***Maternal variables*** |  |  |  |  |  |  |  |  |  |  |  |  |  |  |  |
| **ANC visit** |  |  |  |  |  |  |  |  |  |  |  |  |  |  |  |
| 0-3 |  |  |  |  |  |  | 1.00 |  |  |  |  |  |  |  |  |
| 4+ |  |  |  |  |  |  | 1.63^**^ | 1.46 | 1.82 |  |  |  |  |  |  |
| **Birth attendant** |  |  |  |  |  |  |  |  |  |  |  |  |  |  |  |
| Non-skilled others |  |  |  |  |  |  |  |  |  | 1.00 |  |  |  |  |  |
| Traditional birth attendant |  |  |  |  |  |  |  |  |  | 0.52^*^ | 0.52 | 0.52 |  |  |  |
| Health professional |  |  |  |  |  |  |  |  |  | 1.13 | 1.13 | 1.13 |  |  |  |
| **Decision-making autonomy** |  |  |  |  |  |  |  |  |  |  |  |  |  |  |  |
| High |  |  |  |  |  |  | 1.00 |  |  |  |  |  | 1.00 |  |  |
| Low |  |  |  |  |  |  | 0.88^*^ | 0.80 | 0.97 |  |  |  | 0.67^*^ | 0.46 | 0.96 |
| **Delivery type** |  |  |  |  |  |  |  |  |  |  |  |  |  |  |  |
| Not caesarean | 1.00 |  |  | 1.00 |  |  | 1.00 |  |  | 1.00 |  |  | 1.00 |  |  |
| Cesarean | 0.42^**^ | 0.27 | 0.68 | 0.32^**^ | 0.23 | 0.44 | 0.53^**^ | 0.46 | 0.60 | 0.15^**^ | 0.10 | 0.23 | 0.40^*^ | 0.19 | 0.81 |
| **Gender role attitudes** |  |  |  |  |  |  |  |  |  |  |  |  |  |  |  |
| Nonconforming | 1.00 |  |  | 1.00 |  |  |  |  |  |  |  |  |  |  |  |
| Conforming | 0.87 | 0.74 | 1.03 | 0.84 | 0.68 | 1.02 |  |  |  |  |  |  |  |  |  |
| **Maternal body mass index** |  |  |  |  |  |  |  |  |  |  |  |  |  |  |  |
| Normal |  |  |  |  |  |  |  |  |  |  |  |  | 1.00 |  |  |
| Underweight |  |  |  |  |  |  |  |  |  |  |  |  | 0.70 | 0.39 | 1.26 |
| Overweight |  |  |  |  |  |  |  |  |  |  |  |  | 0.59^*^ | 0.40 | 0.89 |
| **Maternal education** |  |  |  |  |  |  |  |  |  |  |  |  |  |  |  |
| No school |  |  |  |  |  |  | 1.00 |  |  |  |  |  | 1.00 |  |  |
| Primary |  |  |  |  |  |  | 1.05 | 0.89 | 1.24 |  |  |  | 0.93 | 0.53 | 1.63 |
| Secondary+ |  |  |  |  |  |  | 1.19^*^ | 1.05 | 1.35 |  |  |  | 1.43 | 0.89 | 2.31 |
| **Place of delivery** |  |  |  |  |  |  |  |  |  |  |  |  |  |  |  |
| Home | 1.00 |  |  | 1.00 |  |  | 1.00 |  |  | 1.00 |  |  |  |  |  |
| Health facility | 1.19 | 0.99 | 1.42 | 0.76^*^ | 0.58 | 0.98 | 1.41^**^ | 1.22 | 1.63 | 1.80^**^ | 1.18 | 2.76 |  |  |  |
| ***Household variables*** |  |  |  |  |  |  |  |  |  |  |  |  |  |  |  |
| **Caste** |  |  |  |  |  |  |  |  |  |  |  |  |  |  |  |
| *India* |  |  |  |  |  |  |  |  |  |  |  |  |  |  |  |
| Otherwise backwards caste |  |  |  |  |  |  | 1.00 |  |  |  |  |  |  |  |  |
| Scheduled caste |  |  |  |  |  |  | 0.99 | 0.88 | 1.12 |  |  |  |  |  |  |
| Scheduled tribe |  |  |  |  |  |  | 1.15 | 0.98 | 1.35 |  |  |  |  |  |  |
| Other |  |  |  |  |  |  | 0.96 | 0.82 | 1.12 |  |  |  |  |  |  |
| *Nepal* |  |  |  |  |  |  |  |  |  |  |  |  |  |  |  |
| Relatively Advantaged |  |  |  |  |  |  |  |  |  | 1.00 |  |  |  |  |  |
| Relatively Disadvantaged |  |  |  |  |  |  |  |  |  | 0.96 | 0.71 | 1.30 |  |  |  |
| Disadvantaged |  |  |  |  |  |  |  |  |  | 0.87 | 0.60 | 1.26 |  |  |  |
| Other |  |  |  |  |  |  |  |  |  | 0.69^*^ | 0.49 | 0.98 |  |  |  |
| **Ethnicity** |  |  |  |  |  |  |  |  |  |  |  |  |  |  |  |
| *Afghanistan* |  |  |  |  |  |  |  |  |  |  |  |  |  |  |  |
| Pashtun | 1.00 |  |  |  |  |  |  |  |  |  |  |  |  |  |  |
| Tajik | 1.62^**^ | 1.26 | 2.09 |  |  |  |  |  |  |  |  |  |  |  |  |
| Other | 1.52^**^ | 1.21 | 1.91 |  |  |  |  |  |  |  |  |  |  |  |  |
| **Household size (members)** |  |  |  |  |  |  |  |  |  |  |  |  |  |  |  |
| *Afghanistan* |  |  |  |  |  |  |  |  |  |  |  |  |  |  |  |
| <7 | 1.00 |  |  |  |  |  |  |  |  |  |  |  |  |  |  |
| 8-10 | 1.09 | 0.90 | 1.33 |  |  |  |  |  |  |  |  |  |  |  |  |
| 11+ | 1.00 | 0.82 | 1.23 |  |  |  |  |  |  |  |  |  |  |  |  |
| *India* |  |  |  |  |  |  |  |  |  |  |  |  |  |  |  |
| 0-5 |  |  |  |  |  |  | 1.00 |  |  |  |  |  |  |  |  |
| 6-9 |  |  |  |  |  |  | 0.89^*^ | 0.80 | 0.99 |  |  |  |  |  |  |
| 10+ |  |  |  |  |  |  | 0.88 | 0.77 | 1.01 |  |  |  |  |  |  |
| **Residence** |  |  |  |  |  |  |  |  |  |  |  |  |  |  |  |
| Urban |  |  |  |  |  |  | 1.00 |  |  | 1.00 |  |  |  |  |  |
| Rural |  |  |  |  |  |  | 0.86 | 0.74 | 1.00 | 0.87 | 0.67 | 1.13 |  |  |  |
| **Wealth quintile** |  |  |  |  |  |  |  |  |  |  |  |  |  |  |  |
| Richest | 1.00 |  |  |  |  |  | 1.00 |  |  | 1.00 |  |  | 1.00 |  |  |
| Richer | 1.42^*^ | 1.12 | 1.80 |  |  |  | 1.24^*^ | 1.04 | 1.47 | 1.22 | 0.83 | 1.80 | 0.36^*^ | 0.19 | 0.68 |
| Middle | 1.40^*^ | 1.11 | 1.77 |  |  |  | 1.41^**^ | 1.16 | 1.70 | 1.04 | 0.71 | 1.52 | 0.66 | 0.35 | 1.26 |
| Poorer | 1.36^*^ | 1.08 | 1.72 |  |  |  | 1.42^**^ | 1.17 | 1.73 | 1.02 | 0.68 | 1.52 | 0.58 | 0.31 | 1.10 |
| Poorest | 1.48^*^ | 1.16 | 1.88 |  |  |  | 1.46^*^ | 1.17 | 1.81 | 1.45 | 0.95 | 2.21 | 1.11 | 0.54 | 2.31 |

*p<0.05, **p<0.001

Table S.2. Predictors of avoidance of prelacteal feeding (APF) among children 0-23 months who live with their mother.

| Predictors of APF | Afghanistan  2015  (n=10,563) | | | Bangladesh  2014  (n=3,067) | | | India  2015-16  (n=14,241) | | | Nepal  2016  (n=1,923) | | | Pakistan  2013  (n=1,383) | | |
| --- | --- | --- | --- | --- | --- | --- | --- | --- | --- | --- | --- | --- | --- | --- | --- |
|  | OR | 95% CI | | OR | 95% CI | | OR | 95% CI | | OR | 95% CI | | OR | 95% CI | |
| ***Child variables*** |  |  |  |  |  |  |  |  |  |  |  |  |  |  |  |
| **Birth size** |  |  |  |  |  |  |  |  |  |  |  |  |  |  |  |
| Larger than average | 1.00 |  |  |  |  |  | 1.00 |  |  |  |  |  | 1.00 |  |  |
| Average | 0.96 | 0.76 | 1.22 |  |  |  | 0.76^**^ | 0.66 | 0.89 |  |  |  | 0.57^*^ | 0.33 | 0.98 |
| Smaller than average | 0.68^*^ | 0.51 | 0.89 |  |  |  | 0.54^**^ | 0.44 | 0.66 |  |  |  | 0.57 | 0.30 | 1.10 |
| **Postnatal check-up** |  |  |  |  |  |  |  |  |  |  |  |  |  |  |  |
| None | 1.00 |  |  | 1.00 |  |  |  |  |  |  |  |  |  |  |  |
| ≤2 days | 0.71^*^ | 0.55 | 0.90 | 0.69^*^ | 0.55 | 0.85 |  |  |  |  |  |  |  |  |  |
| >2 days | 0.61^**^ | 0.49 | 0.75 | 1.13 | 0.73 | 1.74 |  |  |  |  |  |  |  |  |  |
| ***Maternal variables*** |  |  |  |  |  |  |  |  |  |  |  |  |  |  |  |
| **Birth attendant** |  |  |  |  |  |  |  |  |  |  |  |  |  |  |  |
| Non-skilled others |  |  |  | 1.00 |  |  |  |  |  | 1.00 |  |  |  |  |  |
| Traditional birth attendant |  |  |  | 1.10 | 0.77 | 1.58 |  |  |  | 0.37^*^ | 0.20 | 0.69 |  |  |  |
| Health professional |  |  |  | 1.60 | 1.18 | 2.18 |  |  |  | 1.20 | 0.85 | 1.70 |  |  |  |
| **Current employment** |  |  |  |  |  |  |  |  |  |  |  |  |  |  |  |
| No | 1.00 |  |  |  |  |  |  |  |  | 1.00 |  |  |  |  |  |
| Yes | 0.73 | 0.50 | 1.04 |  |  |  |  |  |  | 1.54^*^ | 1.15 | 2.05 |  |  |  |
| **Decision-making autonomy** |  |  |  |  |  |  |  |  |  |  |  |  |  |  |  |
| High | 1.00 |  |  |  |  |  | 1.00 |  |  |  |  |  |  |  |  |
| Low | 0.82^*^ | 0.68 | 0.99 |  |  |  | 0.83^*^ | 0.74 | 0.94 |  |  |  |  |  |  |
| **Delivery type** |  |  |  |  |  |  |  |  |  |  |  |  |  |  |  |
| Not caesarean |  |  |  | 1.00 |  |  | 1.00 |  |  | 1.00 |  |  | 1.00 |  |  |
| Cesarean |  |  |  | 0.40^**^ | 0.29 | 0.56 | 0.49^**^ | 0.42 | 0.57 | 0.28^**^ | 0.18 | 0.45 | 0.74 | 0.42 | 1.05 |
| **Gender role attitudes** |  |  |  |  |  |  |  |  |  |  |  |  |  |  |  |
| Nonconforming | 1.00 |  |  |  |  |  |  |  |  |  |  |  |  |  |  |
| Conforming | 0.77^*^ | 0.64 | 0.94 |  |  |  |  |  |  |  |  |  |  |  |  |
| **Maternal age (years)** |  |  |  |  |  |  |  |  |  |  |  |  |  |  |  |
| 15-19 |  |  |  |  |  |  |  |  |  | 1.00 |  |  |  |  |  |
| 20-29 |  |  |  |  |  |  |  |  |  | 1.21 | 0.85 | 1.72 |  |  |  |
| 30+ |  |  |  |  |  |  |  |  |  | 1.51 | 0.99 | 2.30 |  |  |  |
| **Maternal body mass index** |  |  |  |  |  |  |  |  |  |  |  |  |  |  |  |
| Normal |  |  |  |  |  |  |  |  |  |  |  |  | 1.00 |  |  |
| Underweight |  |  |  |  |  |  |  |  |  |  |  |  | 1.51 | 0.92 | 2.50 |
| Overweight |  |  |  |  |  |  |  |  |  |  |  |  | 1.10 | 0.76 | 1.58 |
| **Maternal education** |  |  |  |  |  |  |  |  |  |  |  |  |  |  |  |
| No school |  |  |  |  |  |  | 1.00 |  |  | 1.00 |  |  |  |  |  |
| Primary |  |  |  |  |  |  | 1.19 | 0.99 | 1.43 | 1.38 | 0.87 | 2.18 |  |  |  |
| Secondary+ |  |  |  |  |  |  | 1.43^**^ | 1.23 | 1.66 | 1.71^*^ | 1.22 | 2.41 |  |  |  |
| **Place of delivery** |  |  |  |  |  |  |  |  |  |  |  |  |  |  |  |
| Home |  |  |  |  |  |  |  |  |  |  |  |  |  |  |  |
| Health facility |  |  |  |  |  |  |  |  |  |  |  |  |  |  |  |
| ***Household variables*** |  |  |  |  |  |  |  |  |  |  |  |  |  |  |  |
| **Caste** |  |  |  |  |  |  |  |  |  |  |  |  |  |  |  |
| *India* |  |  |  |  |  |  |  |  |  |  |  |  |  |  |  |
| Otherwise backwards caste |  |  |  |  |  |  | 1.00 |  |  |  |  |  |  |  |  |
| Scheduled caste |  |  |  |  |  |  | 1.21^*^ | 1.05 | 1.39 |  |  |  |  |  |  |
| Scheduled tribe |  |  |  |  |  |  | 2.25^**^ | 1.82 | 2.79 |  |  |  |  |  |  |
| Other |  |  |  |  |  |  | 1.09 | 0.93 | 1.27 |  |  |  |  |  |  |
| *Nepal* |  |  |  |  |  |  |  |  |  |  |  |  |  |  |  |
| Relatively Advantaged |  |  |  |  |  |  |  |  |  | 1.00 |  |  |  |  |  |
| Relatively Disadvantaged |  |  |  |  |  |  |  |  |  | 0.71 | 0.50 | 1.00 |  |  |  |
| Disadvantaged |  |  |  |  |  |  |  |  |  | 0.78 | 0.52 | 1.17 |  |  |  |
| Other |  |  |  |  |  |  |  |  |  | 0.64^*^ | 0.45 | 0.91 |  |  |  |
| **Ethnicity** |  |  |  |  |  |  |  |  |  |  |  |  |  |  |  |
| *Afghanistan* |  |  |  |  |  |  |  |  |  |  |  |  |  |  |  |
| Pashtun | 1.00 |  |  |  |  |  |  |  |  |  |  |  |  |  |  |
| Tajik | 1.01 | 0.77 | 1.33 |  |  |  |  |  |  |  |  |  |  |  |  |
| Other | 1.30 | 0.88 | 1.94 |  |  |  |  |  |  |  |  |  |  |  |  |
| **Household size (members)** |  |  |  |  |  |  |  |  |  |  |  |  |  |  |  |
| *India* |  |  |  |  |  |  |  |  |  |  |  |  |  |  |  |
| 0-5 |  |  |  |  |  |  | 1.00 |  |  |  |  |  |  |  |  |
| 6-9 |  |  |  |  |  |  | 0.84^*^ | 0.73 | 0.96 |  |  |  |  |  |  |
| 10+ |  |  |  |  |  |  | 0.69^**^ | 0.59 | 0.81 |  |  |  |  |  |  |
| *Nepal* |  |  |  |  |  |  |  |  |  |  |  |  |  |  |  |
| 0-4 |  |  |  |  |  |  |  |  |  | 1.00 |  |  |  |  |  |
| 5-7 |  |  |  |  |  |  |  |  |  | 0.85 | 0.62 | 1.17 |  |  |  |
| 8+ |  |  |  |  |  |  |  |  |  | 0.73 | 0.52 | 1.02 |  |  |  |
| *Pakistan* |  |  |  |  |  |  |  |  |  |  |  |  |  |  |  |
| 0-6 |  |  |  |  |  |  |  |  |  |  |  |  | 1.00 |  |  |
| 7-11 |  |  |  |  |  |  |  |  |  |  |  |  | 1.11 | 0.77 | 1.61 |
| 11+ |  |  |  |  |  |  |  |  |  |  |  |  | 1.49 | 0.92 | 2.41 |
| **Residence** |  |  |  |  |  |  |  |  |  |  |  |  |  |  |  |
| Urban |  |  |  | 1.00 |  |  |  |  |  |  |  |  |  |  |  |
| Rural |  |  |  | 1.15 | 0.89 | 1.48 |  |  |  |  |  |  |  |  |  |
| **Wealth quintile** |  |  |  |  |  |  |  |  |  |  |  |  |  |  |  |
| Richest |  |  |  |  |  |  | 1.00 |  |  | 1.00 |  |  | 1.00 |  |  |
| Richer |  |  |  |  |  |  | 1.25^*^ | 1.02 | 1.55 | 1.18 | 0.79 | 1.77 | 0.62^*^ | 0.38 | 1.00 |
| Middle |  |  |  |  |  |  | 1.19 | 0.98 | 1.45 | 1.26 | 0.80 | 1.98 | 0.64 | 0.39 | 1.06 |
| Poorer |  |  |  |  |  |  | 1.30^*^ | 1.06 | 1.58 | 1.95^*^ | 1.26 | 3.01 | 0.82 | 0.49 | 1.39 |
| Poorest |  |  |  |  |  |  | 1.07 | 0.86 | 1.33 | 3.60^**^ | 2.04 | 6.35 | 1.57 | 0.89 | 2.75 |

^*^p<0.05, ^**^p<0.001

Table S.3. Predictors of exclusive breastfeeding (EBF) among children 0-5 months who live with their mother.

| Predictors of EBF | Afghanistan  2015  (n=3,026) | | | Bangladesh  2014  (n=651) | | | India  2015-16  (n=3,537) | | | Nepal  2016  (n=443) | | | Pakistan  2013  (n=1,151) | | |
| --- | --- | --- | --- | --- | --- | --- | --- | --- | --- | --- | --- | --- | --- | --- | --- |
|  | OR | 95% CI | | OR | 95% CI | | OR | 95% CI | | OR | 95% CI | | OR | 95% CI | |
| ***Child variables*** |  |  |  |  |  |  |  |  |  |  |  |  |  |  |  |
| **Birth size** |  |  |  |  |  |  |  |  |  |  |  |  |  |  |  |
| Larger than average | 1.00 |  |  |  |  |  |  |  |  |  |  |  |  |  |  |
| Average | 1.03 | 0.67 | 1.57 |  |  |  |  |  |  |  |  |  |  |  |  |
| Smaller than average | 0.55^*^ | 0.35 | 0.86 |  |  |  |  |  |  |  |  |  |  |  |  |
| **Child age (months)** |  |  |  |  |  |  |  |  |  |  |  |  |  |  |  |
| 0-1 | 2.91^**^ | 2.08 | 3.88 | 6.46^**^ | 3.22 | 12.97 | 3.98^**^ | 3.03 | 5.22 | 6.13^**^ | 3.44 | 10.93 | 3.90^**^ | 2.56 | 5.94 |
| 2-3 | 1.55^*^ | 1.17 | 2.06 | 3.27^**^ | 1.77 | 6.04 | 2.30^**^ | 1.85 | 2.87 | 3.93^**^ | 2.15 | 7.18 | 2.06^**^ | 1.39 | 3.05 |
| 4-5 | 1.00 |  |  | 1.00 |  |  | 1.00 |  |  | 1.00 |  |  | 1.00 |  |  |
| **Child sex** |  |  |  |  |  |  |  |  |  |  |  |  |  |  |  |
| Male |  |  |  |  |  |  | 1.00 |  |  |  |  |  |  |  |  |
| Female |  |  |  |  |  |  | 0.80^*^ | 0.66 | 0.97 |  |  |  |  |  |  |
| **Infant health card** |  |  |  |  |  |  |  |  |  |  |  |  |  |  |  |
| No |  |  |  | 1.00 |  |  | 1.00 |  |  |  |  |  |  |  |  |
| Yes |  |  |  | 0.55^*^ | 0.33 | 0.91 | 1.54^*^ | 1.21 | 1.97 |  |  |  |  |  |  |
| **Postnatal check-up** |  |  |  |  |  |  |  |  |  |  |  |  |  |  |  |
| None | 1.00 |  |  |  |  |  |  |  |  |  |  |  |  |  |  |
| ≤2 days | 0.66 | 0.38 | 1.13 |  |  |  |  |  |  |  |  |  |  |  |  |
| >2 days | 0.83 | 0.59 | 1.17 |  |  |  |  |  |  |  |  |  |  |  |  |
| ***Maternal variables*** |  |  |  |  |  |  |  |  |  |  |  |  |  |  |  |
| **ANC visit** |  |  |  |  |  |  |  |  |  |  |  |  |  |  |  |
| 0-3 | 1.00 |  |  |  |  |  |  |  |  |  |  |  |  |  |  |
| 4+ | 0.72 | 0.51 | 1.02 |  |  |  |  |  |  |  |  |  |  |  |  |
| **Birth attendant** |  |  |  |  |  |  |  |  |  |  |  |  |  |  |  |
| Non-skilled others | 1.00 |  |  |  |  |  |  |  |  |  |  |  |  |  |  |
| Traditional birth attendant | 0.43^**^ | 0.29 | 0.63 |  |  |  |  |  |  |  |  |  |  |  |  |
| Health professional | 0.60^*^ | 0.43 | 0.83 |  |  |  |  |  |  |  |  |  |  |  |  |
| **Decision-making autonomy** |  |  |  |  |  |  |  |  |  |  |  |  |  |  |  |
| High |  |  |  |  |  |  |  |  |  | 1.00 |  |  |  |  |  |
| Low |  |  |  |  |  |  |  |  |  | 1.57 | 0.89 | 2.76 |  |  |  |
| **Delivery type** |  |  |  |  |  |  |  |  |  |  |  |  |  |  |  |
| Not caesarean |  |  |  |  |  |  |  |  |  |  |  |  | 1.00 |  |  |
| Caesarean |  |  |  |  |  |  |  |  |  |  |  |  | 0.55^*^ | 0.36 | 0.85 |
| **Gender role attitudes** |  |  |  |  |  |  |  |  |  |  |  |  |  |  |  |
| Nonconforming | 1.00 |  |  |  |  |  |  |  |  |  |  |  |  |  |  |
| Conforming | 0.68^*^ | 0.47 | 0.99 |  |  |  |  |  |  |  |  |  |  |  |  |
| **Maternal age (years)** |  |  |  |  |  |  |  |  |  |  |  |  |  |  |  |
| 15-19 |  |  |  |  |  |  | 1.00 |  |  |  |  |  | 1.00 |  |  |
| 20-29 |  |  |  |  |  |  | 0.62^*^ | 0.42 | 0.94 |  |  |  | 0.54 | 0.28 | 1.05 |
| 30+ |  |  |  |  |  |  | 0.66 | 0.41 | 1.03 |  |  |  | 0.62 | 0.32 | 1.21 |
| ***Household variables*** |  |  |  |  |  |  |  |  |  |  |  |  |  |  |  |
| **Caste** |  |  |  |  |  |  |  |  |  |  |  |  |  |  |  |
| *India* |  |  |  |  |  |  |  |  |  |  |  |  |  |  |  |
| Otherwise backwards caste |  |  |  |  |  |  | 1.00 |  |  |  |  |  |  |  |  |
| Scheduled Caste |  |  |  |  |  |  | 1.14 | 0.90 | 1.46 |  |  |  |  |  |  |
| Scheduled Tribe |  |  |  |  |  |  | 1.39^*^ | 1.04 | 1.86 |  |  |  |  |  |  |
| Other |  |  |  |  |  |  | 0.75 | 0.53 | 1.05 |  |  |  |  |  |  |
| *Nepal* |  |  |  |  |  |  |  |  |  |  |  |  |  |  |  |
| Relatively Advantaged |  |  |  |  |  |  |  |  |  | 1.00 |  |  |  |  |  |
| Relatively Disadvantaged |  |  |  |  |  |  |  |  |  | 0.95 | 0.52 | 1.74 |  |  |  |
| Disadvantaged |  |  |  |  |  |  |  |  |  | 0.55 | 0.27 | 1.14 |  |  |  |
| Others |  |  |  |  |  |  |  |  |  | 0.85 | 0.45 | 1.61 |  |  |  |
| **Ethnicity** |  |  |  |  |  |  |  |  |  |  |  |  |  |  |  |
| *Afghanistan* |  |  |  |  |  |  |  |  |  |  |  |  |  |  |  |
| Pashtun | 1.00 |  |  |  |  |  |  |  |  |  |  |  |  |  |  |
| Tajik | 0.56^**^ | 0.41 | 0.77 |  |  |  |  |  |  |  |  |  |  |  |  |
| Other | 0.39^*^ | 0.28 | 0.56 |  |  |  |  |  |  |  |  |  |  |  |  |
| **Household size (members)** |  |  |  |  |  |  |  |  |  |  |  |  |  |  |  |
| *Bangladesh* |  |  |  |  |  |  |  |  |  |  |  |  |  |  |  |
| 0-4 |  |  |  | 1.00 |  |  |  |  |  |  |  |  |  |  |  |
| 5-8 |  |  |  | 0.47^*^ | 0.27 | 0.84 |  |  |  |  |  |  |  |  |  |
| 9+ |  |  |  | 0.55 | 0.30 | 1.03 |  |  |  |  |  |  |  |  |  |
| **Wealth quintile** |  |  |  |  |  |  |  |  |  |  |  |  |  |  |  |
| Richest | 1.00 |  |  |  |  |  |  |  |  | 1.00 |  |  |  |  |  |
| Richer | 1.31 | 0.88 | 1.94 |  |  |  |  |  |  | 1.17 | 0.53 | 2.59 |  |  |  |
| Middle | 1.12 | 0.76 | 1.66 |  |  |  |  |  |  | 2.17^*^ | 1.04 | 4.53 |  |  |  |
| Poorer | 0.96 | 0.63 | 1.47 |  |  |  |  |  |  | 1.19 | 0.52 | 2.68 |  |  |  |
| Poorest | 1.35 | 0.89 | 2.06 |  |  |  |  |  |  | 2.09 | 0.99 | 4.42 |  |  |  |

^*^p<0.05, ^**^p<0.001

| Table S.4. Predictors of continued breastfeeding (CBF) among children 20-23 months who live with their mother. | | | | | | | | | | | | | | | | |
| --- | --- | --- | --- | --- | --- | --- | --- | --- | --- | --- | --- | --- | --- | --- | --- | --- |
|  | Afghanistan  2015  (n=882)  OR 95% CI | | | Bangladesh  2014  (n=510)  OR 95% CI | | | India  2015-16  (n=2,323)  OR 95% CI | | | Nepal  2016  (n=318)  OR 95% CI | | | Pakistan  2013  (n=396)  OR 95% CI | | |  |
| ***Child variables*** |  |  |  |  |  |  |  |  |  |  |  |  |  |  |  |  |
| **Child sex** |  |  |  |  |  |  |  |  |  |  |  |  |  |  |  |  |
| Male |  |  |  |  |  |  |  |  |  |  |  |  | 1.00 |  |  |  |
| Female |  |  |  |  |  |  |  |  |  |  |  |  | 0.61 | 0.37 | 1.02 |  |
| ***Maternal variables*** |  |  |  |  |  |  |  |  |  |  |  |  |  |  |  |  |
| **Decision-making autonomy** |  |  |  |  |  |  |  |  |  |  |  |  |  |  |  |  |
| High |  |  |  |  |  |  | 1.00 |  |  |  |  |  | 1.00 |  |  |  |
| Low |  |  |  |  |  |  | 0.84 | 0.65 | 1.09 |  |  |  | 0.51^*^ | 0.27 | 0.93 |  |
| **Current employment** |  |  |  |  |  |  |  |  |  |  |  |  |  |  |  |  |
| No |  |  |  |  |  |  |  |  |  | 1.00 |  |  |  |  |  |  |
| Yes |  |  |  |  |  |  |  |  |  | 1.06 | 0.05 | 22.16 |  |  |  |  |
| **Gender role attitudes** |  |  |  |  |  |  |  |  |  |  |  |  |  |  |  |  |
| Nonconforming |  |  |  |  |  |  |  |  |  | 1.00 |  |  |  |  |  |  |
| Conforming |  |  |  |  |  |  |  |  |  | 0.42 | 0.02 | 8.81 |  |  |  |  |
| **Maternal age (years)** |  |  |  |  |  |  |  |  |  |  |  |  |  |  |  |  |
| 15-19 |  |  |  | 1.00 |  |  |  |  |  |  |  |  |  |  |  |  |
| 20-29 |  |  |  | 0.49 | 0.20 | 1.22 |  |  |  |  |  |  |  |  |  |  |
| 30+ |  |  |  | 0.91 | 0.27 | 3.05 |  |  |  |  |  |  |  |  |  |  |
| **Maternal body mass index** |  |  |  |  |  |  |  |  |  |  |  |  |  |  |  |  |
| Normal |  |  |  | 1.00 |  |  | 1.00 |  |  |  |  |  |  |  |  |  |
| Underweight |  |  |  | 1.25 | 0.46 | 3.39 | 1.57^*^ | 1.13 | 2.18 |  |  |  |  |  |  |  |
| Overweight |  |  |  | 0.48 | 0.20 | 1.17 | 0.86 | 0.60 | 1.24 |  |  |  |  |  |  |  |
| **Maternal education** |  |  |  |  |  |  |  |  |  |  |  |  |  |  |  |  |
| No school |  |  |  |  |  |  |  |  |  |  |  |  | 1.00 |  |  |  |
| Primary |  |  |  |  |  |  |  |  |  |  |  |  | 2.36^*^ | 1.03 | 5.42 |  |
| Secondary+ |  |  |  |  |  |  |  |  |  |  |  |  | 1.51 | 0.76 | 3.01 |  |
| **Parity** |  |  |  |  |  |  |  |  |  |  |  |  |  |  |  |  |
| *Afghanistan/Pakistan* |  |  |  |  |  |  |  |  |  |  |  |  |  |  |  |  |
| Low (1-3) | 1.00 |  |  |  |  |  |  |  |  |  |  |  | 1.00 |  |  |  |
| High (4+) | 1.30 | 0.89 | 1.91 |  |  |  |  |  |  |  |  |  | 1.79 | 0.96 | 3.32 |  |
| ***Household variables*** |  |  |  |  |  |  |  |  |  |  |  |  |  |  |  |  |
| **Caste** |  |  |  |  |  |  |  |  |  |  |  |  |  |  |  |  |
| India |  |  |  |  |  |  |  |  |  |  |  |  |  |  |  |  |
| Otherwise backwards caste |  |  |  |  |  |  | 1.00 |  |  |  |  |  |  |  |  |  |
| Scheduled Caste |  |  |  |  |  |  | 1.30 | 0.93 | 1.83 |  |  |  |  |  |  |  |
| Scheduled Tribe |  |  |  |  |  |  | 1.30 | 0.86 | 1.97 |  |  |  |  |  |  |  |
| Other |  |  |  |  |  |  | 1.42 | 1.00 | 2.03 |  |  |  |  |  |  |  |
| **Ethnicity** |  |  |  |  |  |  |  |  |  |  |  |  |  |  |  |  |
| *Afghanistan* |  |  |  |  |  |  |  |  |  |  |  |  |  |  |  |  |
| Pashtun | 1.00 |  |  |  |  |  |  |  |  |  |  |  |  |  |  |  |
| Tajik | 1.20 | 0.77 | 1.88 |  |  |  |  |  |  |  |  |  |  |  |  |  |
| Other | 1.65 | 0.90 | 3.01 |  |  |  |  |  |  |  |  |  |  |  |  |  |
| **Household size (members)** |  |  |  |  |  |  |  |  |  |  |  |  |  |  |  |  |
| *Afghanistan* |  |  |  |  |  |  |  |  |  |  |  |  |  |  |  |  |
| 0-7 | 1.00 |  |  |  |  |  |  |  |  |  |  |  |  |  |  |  |
| 8-10 | 0.64 | 0.39 | 1.07 |  |  |  |  |  |  |  |  |  |  |  |  |  |
| 11+ | 0.98 | 0.59 | 1.64 |  |  |  |  |  |  |  |  |  |  |  |  |  |
| **Residence** |  |  |  |  |  |  |  |  |  |  |  |  |  |  |  |  |
| Urban |  |  |  |  |  |  |  |  |  | 1.00 |  |  |  |  |  |  |
| Rural |  |  |  |  |  |  |  |  |  | 0.76 | 0.05 | 12.58 |  |  |  |  |
| **Wealth quintile** |  |  |  |  |  |  |  |  |  |  |  |  |  |  |  |  |
| Richest |  |  |  | 1.00 |  |  | 1.00 |  |  |  |  |  | 1.00 |  |  |  |
| Richer |  |  |  | 1.47 | 0.56 | 3.86 | 1.23 | 0.79 | 1.92 |  |  |  | 1.23 | 0.54 | 2.76 |  |
| Middle |  |  |  | 1.46 | 0.50 | 4.24 | 1.70 | 1.10 | 2.60 |  |  |  | 3.94^*^ | 1.49 | 10.44 |  |
| Poorer |  |  |  | 3.09 | 0.86 | 11.14 | 2.42^**^ | 1.57 | 3.74 |  |  |  | 3.18^*^ | 1.19 | 8.46 |  |
| Poorest |  |  |  | 2.79 | 0.84 | 9.34 | 3.27^**^ | 2.09 | 5.12 |  |  |  | 5.27^*^ | 1.76 | 15.73 |  |

^*^p<0.05, ^**^p<0.001
